# Asylums in India

**Published:** 1893-02-18

**Authors:** 


### ASYLUMS IN INDIA.

### III.—THE BENGAL PRESIDENCY.

At the five asylums at Cuttack, Dullunda, Berhampore, Dacca, and Patna there is accommodation for 1,069 patients, calculated at 50 superficial feet for each patient, and this capacity will be largely increased when the new buildings at Berhampore are completed. On January 1st, 1891, there were 1,021 patients under treatment, and 213 were admitted or readmitted during the year, giving a total population of 1,234 insane; of these 149 were discharged cured, 31 improved, 3 not improved, 8 "otherwise," and 80 died, leaving 963 in residence at the end of the year. The following table shows the daily average strength at each institution, together with the total number of recoveries, and the percentage of recoveries on the daily average strength:—

| | Daily Average Strength. | Number of Recoveries. | Percentage of Recoveries on Daily Average Strength. |
|---|---|---|---|
| Cuttack | 38·26 | 29 | 75·79 |
| Dullunda | 261·55 | 52 | 19·91 |
| Berhampore | 206·69 | 24 | 11·61 |
| Dacca | 236·87 | 27 | 11·39 |
| Patna | 254·40 | 17 | 6·68 |

The mortality rose from 6·94 in 1890 to [illegible]02 per cent. in 1891, but was still very low. Tak[illegible] the asylums separately, the death-rate was 6·77 at Be[illegible]pore, 7·27 at Dullunda, 7·86 at Patna, 8·86 at Dacca, and [illegible]·68 at Cuttack. The highest percentage of deaths and of recoveries is thus shown by Cuttack, owing undoubtedly to the fact that at Cuttack also there was the smallest average number in confinement. A somewhat elaborate comparison between the death-rate in Bengal and in the other provinces of India is drawn in this report, and also between the death-rate in Bengal and in European asylums, but we doubt if such comparisons are of any practical value, however interesting they may be, whereas the decennial provincial returns as given in this report teach some very important lessons, and moreover bear irresistible testimony to good work being done in the asylums of Bengal.

The total expenditure under all heads was Rs.94,780 12a. 1p., against Rs.90,374 15a. 4p. in 1890, and the total average yearly cost was Rs.95 0a. 5p., against Rs.89 11a. 6p. in the previous year; but deducting the amount received from paying patients, and the public works charges, the average stands at Rs.82 3a. 7p., as compared with Rs.79 2a. 2p. This still includes rates and taxes, and if this is deducted the average cost per lunatic is reduced to Rs.77 13a. 8p., against Rs.76 2a. in 1890. No less than Rs.2,454 was paid as water-tax at Dacca in the year under review, and if this be excluded again the average cost for each lunatic in 1891 stands at Rs.75 6a. 4p.

In addition to these institutions there is a European asylum at Bhowanipore with accommodation for 42 patients—23 males and 19 females. On January 1st there were 34 patients in residence, and 16 were admitted during the year, giving a total population of 50. Of these 3 were cured, 7 died, and 5 were transferred to other places, so that 35 remained under treatment at the end of the year. The total expenditure for the year was Rs.25,370, which, with an average daily number of 37 inmates, gives an average yearly cost of Rs.686 for each lunatic; deducting payments by friends of paying patients, the average yearly cost of each lunatic stood at Rs.420. The income was made up of Rs.15,531 contributed from Government, and Rs.9,839 received from the paying patients. There is a suggestion now under consideration of closing this asylum altogether, and transferring the patients to Dullunda.

"The thanks of the Lieutenant-Governor are due to Dr. Hilson for this report." We make this quotation with pleasure, for we consider they are well earned by an exceptionally clear and straightforward statement of affairs.

